# Fabricating biomimetic materials with ice‐templating for biomedical applications

**DOI:** 10.1002/SMMD.20230017

**Published:** 2023-07-05

**Authors:** Xiang Lin, Lu Fan, Li Wang, Anne M. Filppula, Yunru Yu, Hongbo Zhang

**Affiliations:** ^1^ Pharmaceutical Sciences Laboratory Åbo Akademi University Turku Finland; ^2^ Turku Bioscience Centre University of Turku and Åbo Akademi University Turku Finland

**Keywords:** biomedical application, biomimetic materials, ice‐template, patterning, structural materials

## Abstract

The proper organization of cells and tissues is essential for their functionalization in living organisms. To create materials that mimic natural structures, researchers have developed techniques such as patterning, templating, and printing. Although these techniques own several advantages, these processes still involve complexity, are time‐consuming, and have high cost. To better simulate natural materials with micro/nanostructures that have evolved for millions of years, the use of ice templates has emerged as a promising method for producing biomimetic materials more efficiently. This article explores the historical approaches taken to produce traditional biomimetic structural biomaterials and delves into the principles underlying the ice‐template method and their various applications in the creation of biomimetic materials. It also discusses the most recent biomedical uses of biomimetic materials created via ice templates, including porous microcarriers, tissue engineering scaffolds, and smart materials. Finally, the challenges and potential of current ice‐template technology are analyzed.

## Graphical abstract

Creating materials that mimic natural structures is challenging due to the complexity, time, and cost involved in techniques such as patterning, templating, and printing. The use of ice templates has emerged as a promising method for producing biomimetic materials more efficiently. This review explores the historical approaches and principles underlying the ice‐template method, recent biomedical uses, and analyzes its challenges as well as future potential.

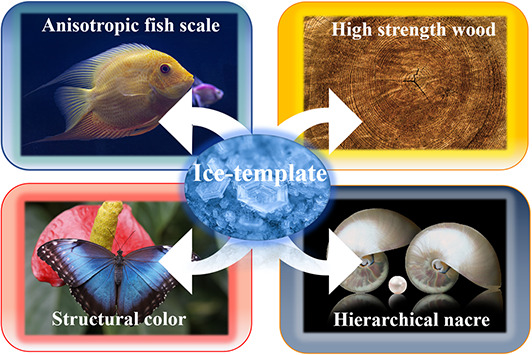

**Key points**

- The historical methods employed in the production of traditional biomimetic structural biomaterials were discussed.
- The fundamental principles of the ice‐template method are discussed.
- Introduced the various biomedical applications of biomimetic materials created through ice templates.
- Addressed the challenges and potential of the current ice‐template technology.

## 1 INTRODUCTION

In the realm of life, from the humblest single‐celled beings to the intricate tapestry of multicellular species, cellular and tissue organization, like humans, weaves an essential pattern that serves vital purposes.^1^ Within this intricately woven fabric, numerous tissues, such as muscles,^2^ blood vessels,^3^ and nerves,^4^ boast elaborate and well‐ordered cellular configurations, forming the foundation for their functionality and resilience. To emulate such remarkable patterns and structures in engineered materials, scientists often turn to the bountiful treasury of nature's masterful designs.^5^ Nature's repertoire of complex and elegant architectures inspires several approaches, such as patterning, templating, and printing, which are frequently employed to fabricate materials that mimic these natural structures. By harnessing these techniques, scientists seek to replicate the remarkable properties and functionalities observed in biological systems.^6^ While biomimetic materials present numerous benefits, such as biocompatibility, robustness, and the capacity to merge with the human body, they also face some limitations in production.^6^, ^7^ For example, their fabrication can be intricate, time‐consuming, and costly, thereby restricting their accessibility and practical applications.^8^ Moreover, natural materials with micro/nano structures have blossomed over millions of years and bear complex and enigmatic properties.^9^

Micro‐nanofabrication techniques grant precise control over material structure and properties, which is crucial for creating biomimetic materials that echo their natural counterparts.^10^ Thereby, materials crafted by mechanical micromachining, lasers, exposure etching, and nanoimprinting possess innovative structures and can attain exceptional properties of some natural materials by emulating nature's blueprint.^11^ However, nanotechnology preparation can be expensive, and replicating natural materials' complex hierarchical structures in synthetic materials poses a challenge.^12^ For instance, bone boasts a hierarchical structure composed of collagen fibers, mineral crystals, and water, which endows it with strength and flexibility.^13^ However, simulating such elaborate structures in synthetic materials is no small feat.^14^ To date, ice template technology has become a more effective method for preparing biomimetic materials.^15^ The ice‐template technique employs ice as a template or mold to create materials with specific structures. This preparation technique enables easy control over the resulting material structure, enabling precise tuning of its properties. The method also allows for large‐scale preparation, satisfying the demand for extensive production. The resulting biomimetic material displays a novel structure and can acquire the remarkable properties of certain natural materials by mimicking nature's design.

In summary, employing ice‐template technology to create biomimetic structural biomaterials offers an enthralling prospect. These traits make them an ideal choice for applications such as drug delivery, tissue engineering, and bodily implants. This article explores the historical strategies used to generate traditional biomimetic structural biomaterials, delves into the principles underpinning the ice‐template method, and examines its diverse applications in crafting biomimetic materials. Furthermore, it discusses the latest biomedical uses of biomimetic materials fashioned through ice templates, including bone tissue engineering, cartilage repair, and wound healing. Lastly, the challenges and potential of current ice‐template technology are scrutinized.

## 2 TRADITIONAL STRATEGIES FOR BIOMIMETIC MATERIALS

**FIGURE 1:**
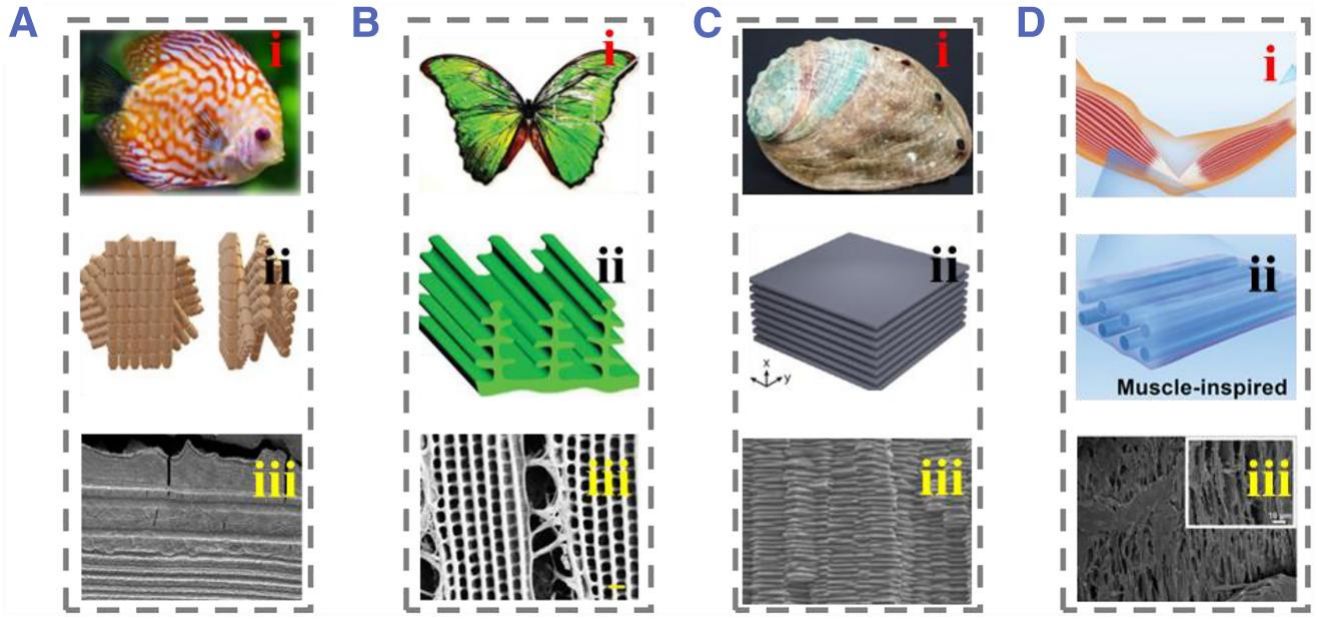
(A) (i) Swimming fish. Reproduced under terms of the CC‐BY license.^24^ Copyright 2019, The Authors, published by MDPI. (ii) Schematic diagram of the layered structure of fish scale. Reproduced with permission.^16^ Copyright 2020, Elsevier. (iii) Cross‐sectional image of fish scale. Reproduced with permission.^16^ Copyright 2020, Elsevier. (B) (i) Colorful butterfly. (ii) Schematic diagram of the upper structure of butterfly wings. (iii) SEM photo of butterfly wings. Reproduced with permission.^17^ Copyright 2019, John Wiley and Sons. (C) (i) Optical image of the nacre, (ii) mechanical composite, and (iii) SEM image of the large area lamb structure. Reproduced with permission.^18^ Copyright 2022, American Chemical Society. (D) (i) Schematic diagram of human arm muscles. (ii) Muscle inspired orientation structure. (iii) The SEM image of the cross‐section view of the conductive hydrogel. Reproduced with permission.^19^ Copyright 2021, John Wiley and Sons. SEM, scanning electron microscopy.

In the evolutionary process of nature, many organisms have mastered the manufacturing of natural biomaterials, which surpass synthetic materials in many aspects, such as fish scales,^16^ butterfly wings,^17^ shells,^18^ mammalian muscle fibers,^19^ and wood^20^ (Figure 1). Typical anisotropic scales commonly found on bony fish provide protection while maintaining the flexibility and mobility of the fish.^21^ The outer layer is composed of highly mineralized woven fibers covered with extremely thin and discontinuous mineral layers. The inner layer is a highly ordered “plywood‐like” structure composed of stacked collagen sheets, and these synergistic oriented structures make the fish scales excellent dermal armor. The unique mechanical properties of the nacre layer are also attributed to its complex layered structure. In addition, many tissues, including muscles, vascular tissue, and nerves, exhibit organized cellular arrangements. It is worth noting the importance of directional cell arrangement.^22^ For example, the appropriate arrangement of heart cells is the basis for heart contraction.^23^ Similarly, the neurite of rat dorsal root ganglion neurons cultured on arrayed nanofibers is longer than that cultured on randomly oriented nanofibers and has more directional characteristics of original neural tissue.

In early research on cell migration, orientation structures have been employed as an effective tool for examining cellular behavior.^23^, ^25^ Numerous studies have explored cell migration and other behaviors by exploiting the similarities between micropattern matrices and tissue structures.^26^ Initially used in microelectronics, micropatterns were later applied in biology. When cultured on these micropatterns, cell migration patterns can be categorized into alignment, restriction, or a combination of both.^27^ On oriented structural materials, the direction of the ravines usually governs cell migration.

Photolithography‐based polyvinyl alcohol (PVA) micropatterns can be utilized to investigate the relationship between mesenchymal stem cell (MSC) differentiation and the underlying substrate.^28^ However, the photolithography method necessitates costly facilities and cleanroom environments for micropattern production, making it challenging to disseminate within the biological field. Alternatively, soft lithography technology employs elastomeric materials, such as poly(dimethylsiloxane) (PDMS), to generate micropatterns due to its straightforward surface modification process, uncomplicated biological fabrication process, and ease of scaling up. Moreover, laser technology and inkjet printing technology can also be employed to fabricate high‐precision patterned structures.^29^ Although these manufacturing techniques satisfy biological requirements in terms of accuracy, they primarily face limitations in material selection, such as the inability of micro‐mode structures to incorporate extracellular matrix components like proteins or growth factors.

Electrospinning technology is an engineering technique that allows precise orientation control at the nanoscale.^30^ A high electrostatic force is applied to the needle of a syringe, causing polymer droplets to form conical shapes, known as Taylor cones, due to the aggregation of charges. In the past, nanofibers sprayed onto a receiver were randomly oriented. However, by enhancing the design of the receiver, directionally structured materials can be obtained. A rotating collection device can continuously elongate and maintain the parallel arrangement of fibers during nanofiber collection. Typically, the spinning speed must align with the rotational speed. For instance, Li et al. assembled Ti_3_C_2_T_x_ fibers with high wafer orientation through a wet spinning process, controlling the spinning speed and spinneret morphology.^31^ The development of living biomaterials is advancing rapidly and has the potential to revolutionize tissue engineering. Living cell fiber biomaterials can be precisely controlled to produce desired biological outputs like cell growth and differentiation.^32^ Xu et al. have made significant strides in this field by using collagen self‐assembly and micro‐sol electrospinning techniques to create targeted living fiber bundles. These fiber bundles have been shown to enhance the transplantation rate of early inflammatory stem cells, which could be critical in treating certain types of injuries or diseases. Additionally, the fiber bundles can indirectly boost the dynamic regulation of stem cells during inflammation, further increasing their potential as a therapeutic tool. With continued progress in this area, living biomaterials have the potential to transform the field of regenerative medicine and greatly improve patient outcomes.^33^

## 3 BIOMIMETIC MATERIALS BASED ON ICE TEMPLATE TECHNOLOGY

**FIGURE 2:**
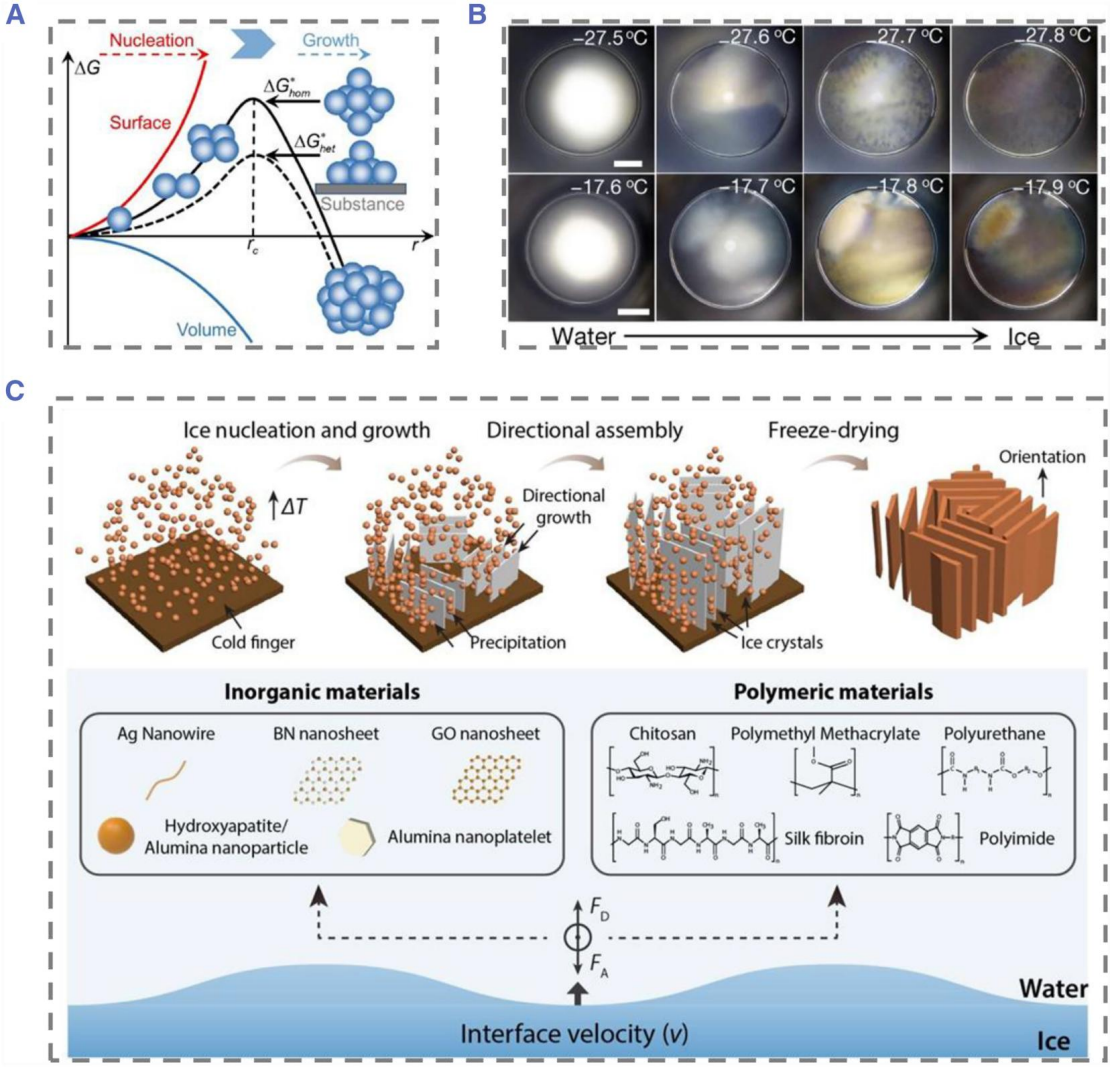
(A) Schematic diagram of classical nucleation theory. Reproduced with permission.^34^ Copyright 2023, Elsevier. (B) Optical microscope images of a typical freezing process of water droplets containing graphene oxide (GOs) with an average lateral size of 8 nm (upper row) and 11 nm (lower row) when the temperature decreases at a constant cooling rate. Reproduced with permission.^35^ Copyright 2019, The Authors, published by Springer Nature. (C) The mechanism of ice template technology and various components assembled at the directionally grown ice‐water interface. Reproduced with permission.^39^ Copyright 2022, American Chemical Society.

Nucleation is the initial and critical process of crystal formation. A century ago, Gibbs and colleagues proposed the “classical nucleation theory” based on thermodynamic principles (Figure 2A).^34^ This theory posits that a phase transition necessitates a single‐step nucleation process, corresponding to the surmounting of a free energy barrier. For instance, in supercooled water, small ice nuclei form, and the phase transition can spontaneously occur only when the resulting ice nuclei, with the final ice structure, accidently surpass the critical size—meaning the system has crossed the free energy barrier associated with the critical ice nuclei. Recently, Bai and co‐authors utilized graphite oxide nanosheets to experimentally confirm the existence of critical ice nuclei and the temperature dependence of their size (Figure 2B).^35^ Ice nuclei serve as the foundation for ice crystal growth; as the polymer solution cools, material accumulates around the expanding ice crystals. The ice crystal formation can be managed through several factors, including solute concentration, solution viscosity, cooling rate, cooling direction, and freezing rate. These factors are critical to consider when dealing with materials that are sensitive to ice formation, such as biological samples or certain food products.^36^ By manipulating these factors, it is possible to control the size, shape, and distribution of ice crystals within a material, which can significantly affect its overall quality and stability.

### 3.1 Ice template technology

Ice templates are a technique that uses ice to introduce porosity into a material solution, suspension, or slurry. As solute particles concentrate around growing ice crystals and the sample freezes, a negative cast of the original ice crystal creates a pore morphology. The direction of cooling can control the voids generated, with multidirectional freezing creating random isotropic ice crystals and unidirectional freezing producing uniformly aligned ones. The rate of solidification affects the ice crystal and pore size, and can be controlled by adjusting the cooling rate, material concentration, and viscosity (Figure 2C).^37^

Ice crystals and suspended materials interact in complex ways, but can be simplified as displacement or entrapment. The solidification rate determines whether displacement or capture occurs. Freezing rate and solution viscosity affect ice crystal shape and resistance to growth, which determines the interaction with the polymer material.^38^ The critical velocity is the key variable that determines displacement and capture:

$$v_{c}={\frac{∆\sigma_{0}d}{3\eta r}\;\left(\frac{a_{0}}{d}\right)}^{n}$$

where *η* represents the viscosity of the solutions, *r* represents the particle radius, ∆*σ*_0_ represents the free energy, *a*_0_ represents average molecular distance, *d* represents the distance between the particle and growing ice, and *n* represents a constant.

### 3.2 Create porous structure with ice template

Ice template technology is a simple and highly controllable method for preparing porous structures, offering extensive application potential. One of its major advantages lies in the continuous pore network present in the resulting porous materials, allowing for the achievement of a significantly large specific surface area. This characteristic is particularly beneficial in various fields, including catalysis, adsorption, and energy storage, as it provides abundant reactive sites and enhanced adsorption/release capabilities. Moreover, the ice template technology enables the creation of porous structural materials that exhibit biocompatibility. This feature is of utmost importance in applications such as tissue engineering, drug delivery, and biosensing.

The pore structure of these porous materials can effectively mimic the microstructure of biological tissues, facilitating cell attachment and growth. Notably, ice template‐based fabrication of microporous polymeric materials has showcased remarkable potential across a range of biomedical applications, encompassing flexible electronic devices, tissue engineering scaffolds, on‐demand delivery platforms, and biosensors.^40^ Initially, porous materials were mainly applied in drug delivery systems for diverse medical purposes. However, as the research advanced, it became apparent that pore morphology is a critical factor in determining the effectiveness of biomaterials for different applications. This is because pore morphology directly influences material transport, cell deposition, and immune response; these abilities constructed the overall performance of the scaffold. The ice template method can adjust the pore morphology by controlling the growth conditions and manipulating the ice crystal formation. The ice template method offers advantages in adjusting the pore morphology by controlling freezing rates, ice crystal nucleation, and template design. These advantages include tunable pore size and shape, highly ordered pore structures, versatility, compatibility, and reproducibility.

In the context of complex tissue structures like neuronal, bone, and cardiac tissue, immune responses can be modulated by controlling the pore size and stiffness of the biomaterials.^41^ This allows for better integration of the biomaterial into the host tissue, resulting in enhanced functionality and decreased likelihood of adverse reactions. Furthermore, pore morphology plays a significant role in the immune response to porous scaffolds used in allogeneic tissue‐engineering implants. As cells infiltrate and organize within these implants, the immune response is affected by the features of the pores, such as size, shape, and distribution. The importance of pore morphology in microporous polymeric materials is now well understood, leading to a more targeted approach in their design.^42^ This approach ensures that the resulting biomaterial is optimized for its intended application, creating new possibilities for the use of these materials in various fields of medicine. As a result, more effective and safer medical devices, implants, and tissue engineering structures can be developed.

### 3.3 Unidirectional freezing

The unidirectional freezing process involves the growth of ice toward a controlled thermal gradient, determined by the cooling rate and direction. This results in preferential ice growth in the direction of the applied field, forming pores that vary in size based on the cooling rate.^43^ The template is subjected to controlled freezing in a unidirectional manner, typically from one side or along a specific axis. This freezing process is carefully controlled to allow ice crystals to grow in a specific direction, guided by the template's physical properties and freezing conditions. A suitable template is chosen based on the desired biomimetic structure. The template can be a solution, suspension, or gel containing the material or materials of interest. Smaller pores are created with faster cooling rates, while larger pores form at slower cooling rates. One of the challenges with unidirectional freezing is the lack of standardized settings, requiring custom setups for each application. Currently, there is no standardized unidirectional freezing setup available; for research on freezing, researchers often rely on manually built freezing platforms that lack uniformity.

**FIGURE 3:**
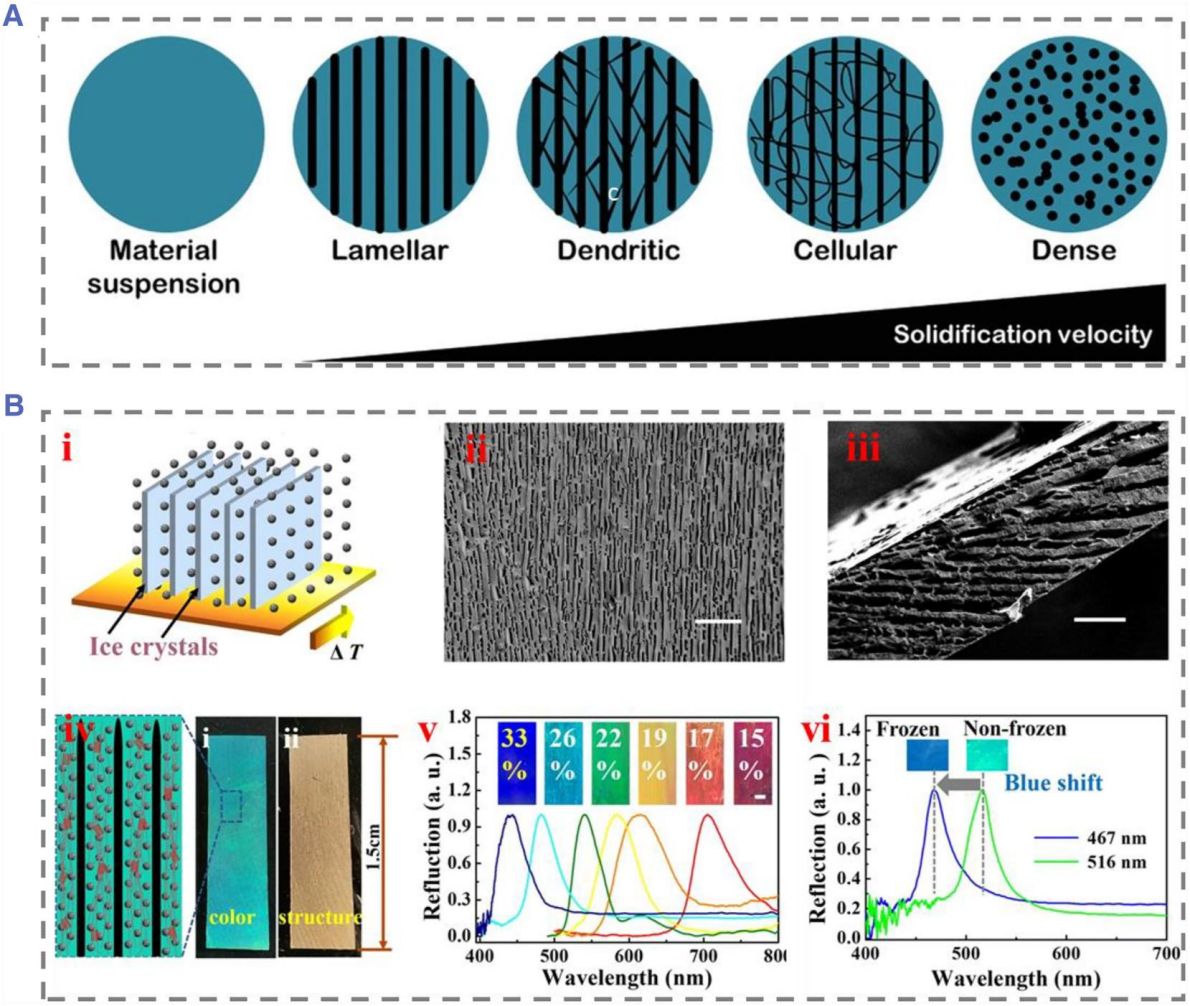
(A) Different ice crystal forms in unidirectional freezing, from independent uniaxial arranged layered ice crystals to dense ice crystal networks. Reproduced with permission.^42^ Copyright 2021, Royal Society of Chemistry. (B) (i) The fabrication process of nanoparticle assembly. (ii, iii) The SEM photos of unidirectional ice template patch. (iv) The images of the resultant color and structures. (v) The wavelength changes of directional frozen films with various concentrations of nanoparticle solutions. (vi) The wavelength changes before frozen and after frozen. Reproduced under terms of the CC‐BY license.^44^ Copyright 2022, The Authors, published by Springer Nature. SEM, scanning electron microscopy.

Generally speaking, the platform should have electric controlled cooling elements and power sources for controlling power. The pore morphology in unidirectional freezing can take various forms (Figure 3A).^42^ The solidification rate is influenced by the cooling rate and material properties, which in turn affect the ice formation and the displacement of suspended matter. The cooling element rate also plays a crucial role in determining solidification and pore morphology as it distributes thermal gradients throughout the sample. Our groups developed a freeze‐derived heterostructured colored hydrogel for information encryption and decryption (Figure 3B).^44^ The space‐occupancy effect of ice crystals has been found to be useful in adjusting the distance of non‐close‐packed colloidal crystal nanoparticles, resulting in a reflection wavelength shift in the frozen region. With control over the growth of ice crystals and photopolymerization, it is possible to create structurally colored hydrogels with specific structures and morphologies. By taking advantage of this technique, it is possible to tailor the properties of these hydrogels to suit different applications in materials science and biomedicine.^45^ This process can also create heterostructured multi‐compartment bodies and multicolored design patterns by changing the cryoregion design. Ongoing research into the relationship between pore morphology and biomaterial properties will undoubtedly lead to further developments in this critical research area, ultimately benefiting patients and healthcare providers.

### 3.4 Multi‐directional freezing

**FIGURE 4:**
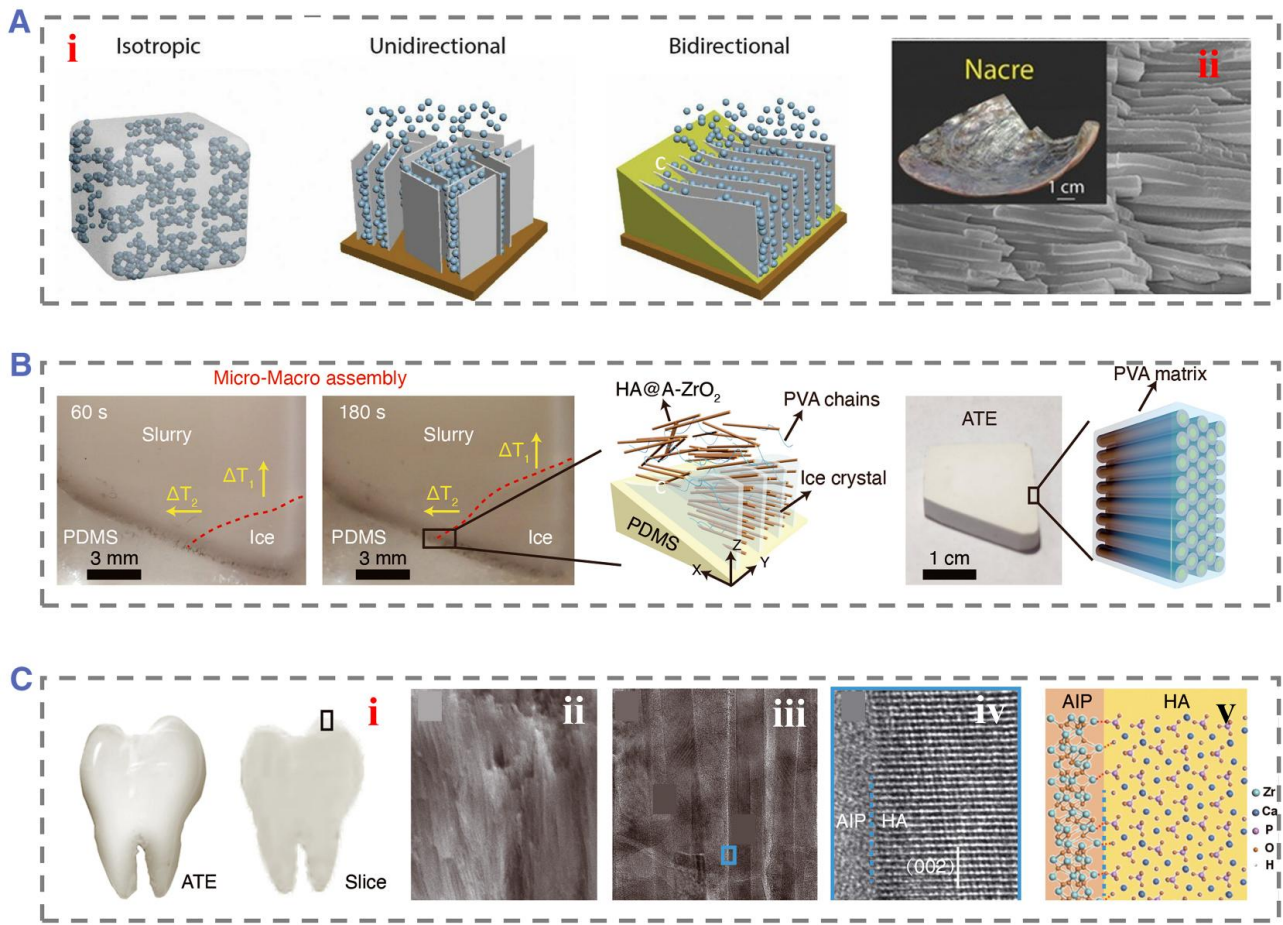
(A) Various (i) directional freezing technology. Isotropic freeze casting, unidirectional freeze casting, and bidirectional freeze casting. Reproduced with permission.^48^ Copyright 2020, Elsevier. (ii) The nacre layer and the SEM image. (B) Schematic diagram of the micro‐ and macro‐assembly of nanowires coupled with polyvinyl alcohol (PVA). Reproduced with permission.^49^ Copyright 2022, The Authors, published by AAAS. (C) (i) Optical photographs of ATE and a sectional view. (ii) SEM photos of the ATE materials. (iii) TEM image of the ATE. (iv) The magnified images of (iii). (v) Illustration of HA as well as amorphous ZrO_2_. Reproduced with permission.^49^ Copyright 2022, The Authors, published by AAAS.

Nacre has a unique structure of layered aragonite flakes bonded with biopolymers that results in exceptional strength and toughness. To fabricate nacre‐mimicking materials, various techniques have been developed, such as layer‐by‐layer assembly, vacuum filtration, 3D printing, and bidirectional freeze casting, which has gained significant attention due to its scalability and eco‐friendliness.^46^ During bidirectional freeze casting, a temperature gradient is generated in both the vertical and horizontal directions due to the low thermal conductivity of the PDMS wedge covering the cold surface.^47^ As a result, ice crystals nucleate exclusively on the lowest side of the wedge and grow preferentially along the vertical and horizontal temperature gradients, forming long‐range single‐domain layered structures. Nacre‐mimicking composites with excellent mechanical properties were fabricated using bidirectional freezing, which involved preparing hydroxyapatite (HA) scaffolds with long‐range aligned layered structures and densifying them further (Figure 4A).^48^ The composites were formed by infiltrating polymethylmethacrylate (PMMA) into the HA scaffolds, resulting in a high ceramic content of up to 75–85 vol%, which could be adjusted by pressing the scaffold to different thicknesses. These composites exhibit a high modulus and a high flexural strength similar to cortical bone.

Fabrication of 3D scaffolds that replicate the complex hierarchical architecture of native tissues is a challenging task. However, regenerative scaffolds with good architectural and physiomechanical properties can improve the engineered tissue function at the cellular and tissue levels. Inspired by enamel, the hardest biological material in the human body, Zhao et al. designed an enamel mimic consisting of HA nanowires using a scalable bidirectional cryo‐arrangement (Figure 4B). The resulting nanocomposites exhibit high stiffness, hardness, strength, viscoelasticity, and toughness, exceeding the properties of enamel and the previously fabricated bulk enamel materials. The presence of amorphous intercrystalline phase, polymer confinement, and strong interfacial adhesion are required for high mechanical properties (Figure 4C).^49^ This multiscale design is suitable for the scalable production of high‐performance materials.

### 3.5 Others

**FIGURE 5:**
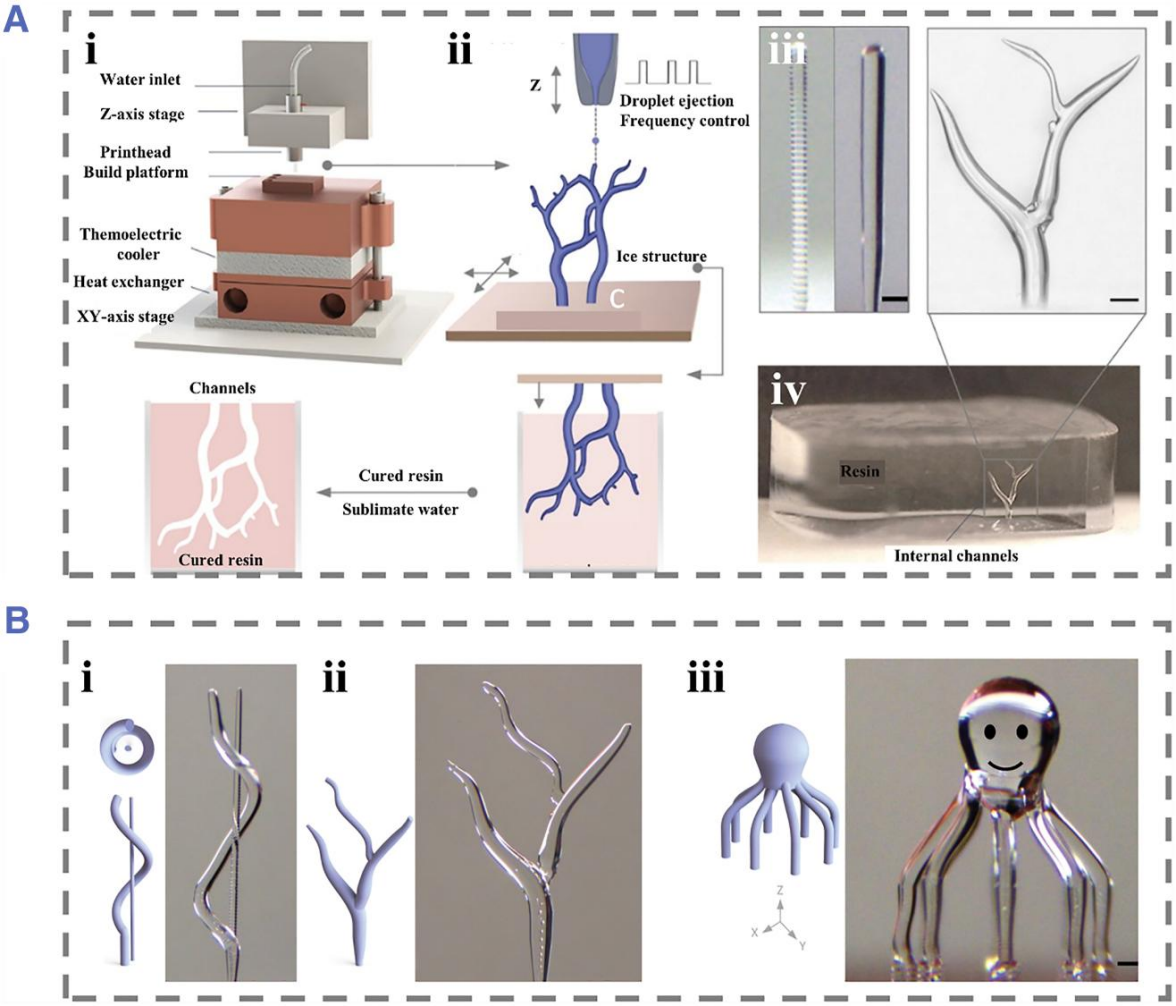
(A) Freeform ice printing. The planar motion during the (i, ii) construction phase is synchronized with droplet discharge to print complex ice geometries, allowing for the printing of tree‐shaped geometric shapes with smooth surfaces and continuous diameter variations with smooth transitions. (iii) Different frequencies lead to different layered geometry. (iv) The ability to accurately capture microscale features of ice templates using reverse molding technology. Reproduced under terms of the CC‐BY license.^50^ Copyright 2022, The Authors, published by John Wiley and Sons. (B) (i, ii, iii) D‐ICE printing of complex 3D geometric shapes with microscale features and smooth walls. Reproduced under terms of the CC‐BY license.^50^ Copyright 2022, The Authors, published by John Wiley and Sons.

The 3D ice printing process is a unique free‐form 3D ice printing technique that can create intricate ice structures with an impressive microscale resolution. Garg et al. have developed an innovative approach for the high‐speed and complex 3D printing of ice structures with smooth surfaces, branched geometries, and continuously changing circular cross‐sections, without the need for support structures or layer‐by‐layer construction (Figure 5A).^50^ The ideal size and resolution of the printed ice features are determined by adjusting the droplet deposition frequency and the *X*–*Y* motion of the high‐precision motion system. To create smooth ice structures with various shapes, such as inclined, branched, layered, and straight geometries, researchers conducted experimental studies to identify the optimal printing path, motion stage velocity, and droplet frequency. Interestingly, even when the *X*–*Y* stage remains stationary, knowing the series before freezing enables rapid and continuous alterations to the size of the printed features. To showcase this groundbreaking technique, several sample 3D ice structures have been printed (Figure 5B).^50^ One exciting application of this free‐form 3D ice printing technology is the creation of sacrificial ice templates with well‐defined internal features.

## 4 BIOMEDICAL APPLICATIONS

Ice template biomimetic materials, which draw inspiration from the captivating beauty and intricacy of natural ice formations, have shown immense potential in a range of biomedical applications.^15^, ^51^ These materials are designed to mimic the unique properties of ice, such as its high surface area and porous structure, resulting in a versatile and effective platform for various therapeutic interventions.^15c^ One of the key applications of these biomimetic materials lies in the realm of tissue engineering. By serving as a scaffold, they create a supportive and conducive environment for cells to grow, proliferate, and ultimately, regenerate damaged tissues.^15d^ The scaffold's intricate architecture is tailored to the specific needs of each application, encouraging cell adhesion and providing ample space for new tissue formation. This attribute has made ice template biomimetic materials indispensable in the quest to engineer functional and healthy tissue constructs.

### 4.1 Porous microcarrier

A primary advantage of ice‐templated biomimetic materials lies in their capacity to emulate the intricate architecture of native tissue.^52^ Such materials can possess interconnected pore networks, facilitating nutrient and waste exchange while providing surfaces conducive to cellular attachment and proliferation.^53^ Additionally, these scaffolds can be functionalized with various biomolecules, including growth factors or extracellular matrix components, to augment their biological properties. Porous microspheres are usually prepared from microfluidic emulsion systems.^54^ The combination of microfluidics technology with the ice template method can provide a powerful platform for precise control and fabrication of complex hierarchical structures with well‐defined features. The formed droplets are cross‐linked by physically or chemically induced methods, resulting in monodisperse microfluidic hydrogel microspheres.^55^ In a specific region of the microfluidic device, the controlled flow of the template solution is subjected to freezing conditions to induce ice templating. This can be achieved by introducing a temperature gradient or a cooling mechanism at the desired location. This microfluidic approach offers several advantages over other microsphere preparation techniques. For example, the size range of hydrogel microspheres is tunable due to the tunable flow rate and length scale of microfluidic devices. In addition, the microfluidic emulsion system allows the rapid preparation of microspheres, meeting high‐yield production requirements.^56^ Copyright 2022, Wiley.

**FIGURE 6:**
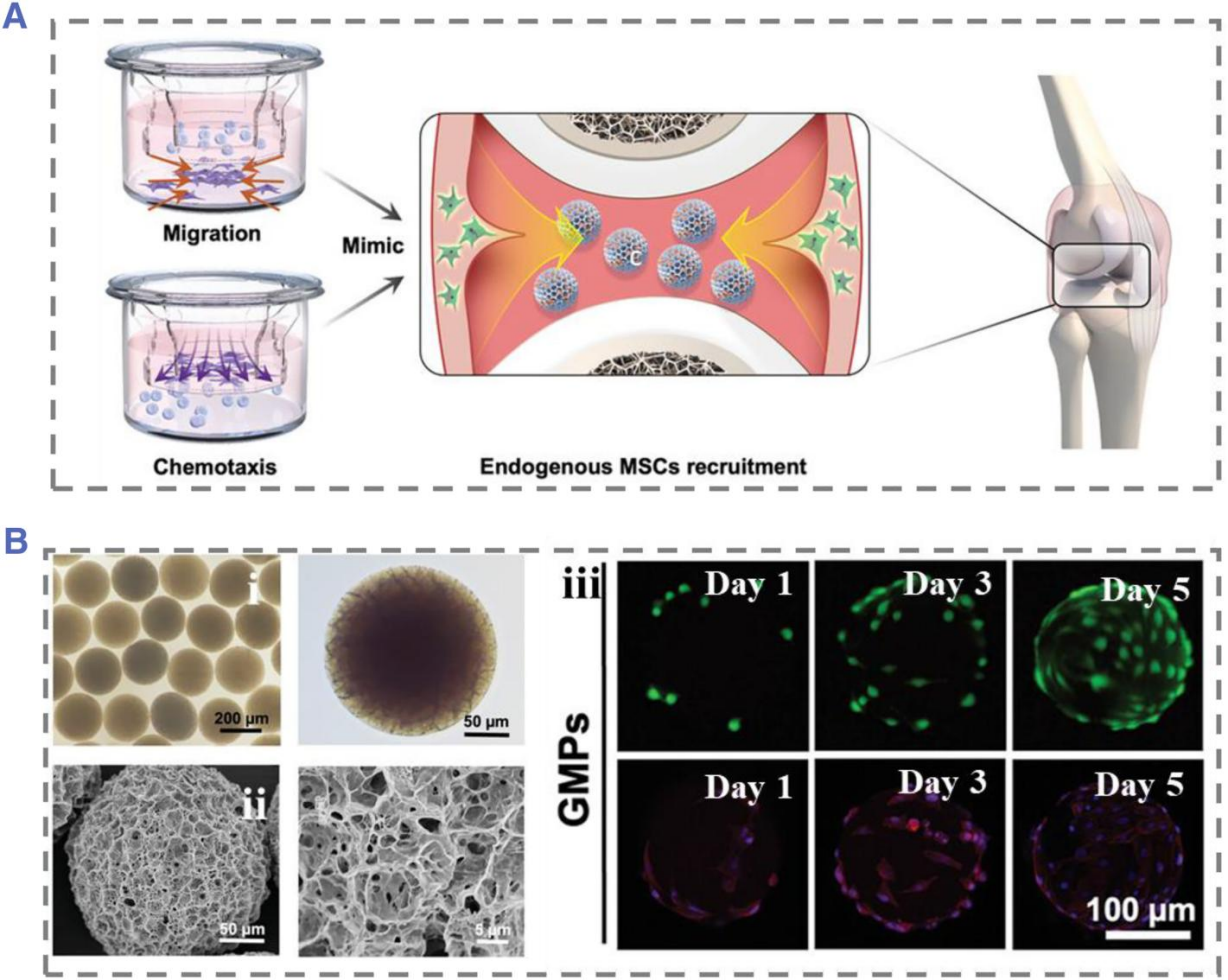
(A) Schematic diagram indicating cell migration and chemotaxis measurement. Reproduced with permission.^57^ Copyright 2023, John Wiley and Sons. (B) (i) Image and magnification field of microspheres. (ii) SEM photos and magnifications of microspheres. (iii) Representative fluorescence images of MSCs cultured with microspheres and characterized by live/dead staining as well as cytoskeleton/nucleus staining. Reproduced with permission.^57^ Copyright 2023, John Wiley and Sons. MSC, mesenchymal stem cell; SEM, scanning electron microscopy.

Cui et al. developed an innovative method to fabricate injectable porous hydrogel microspheres with enduring paracrine activity by incorporating platelet‐derived growth factor and exogenous MSCs into gelatin methacrylate hydrogel microspheres (Figure 6A).^57^ These microcarriers exhibited exceptional mechanical properties and readily adhered and proliferated on the microspheres, promoting interactions between the extracellular matrix and cells to enhance paracrine effects (Figure 6B).^57^ The sustained release of growth factors from the microspheres recruited endogenous MSCs, thereby prolonging the paracrine activity of living microcarriers. These microcarriers demonstrated superior secretory properties and anti‐inflammatory efficacy, potentially mitigating osteoarthritis progression through the promotion of adherent microenvironment and the synergistic effects of exogenous and endogenous MSCs. Both in vitro and in vivo experiments showed promising results, highlighting the potential of this innovative approach for developing functional materials with diverse applications in the field of regenerative medicine.

### 4.2 Smart biomimetic materials

**FIGURE 7:**
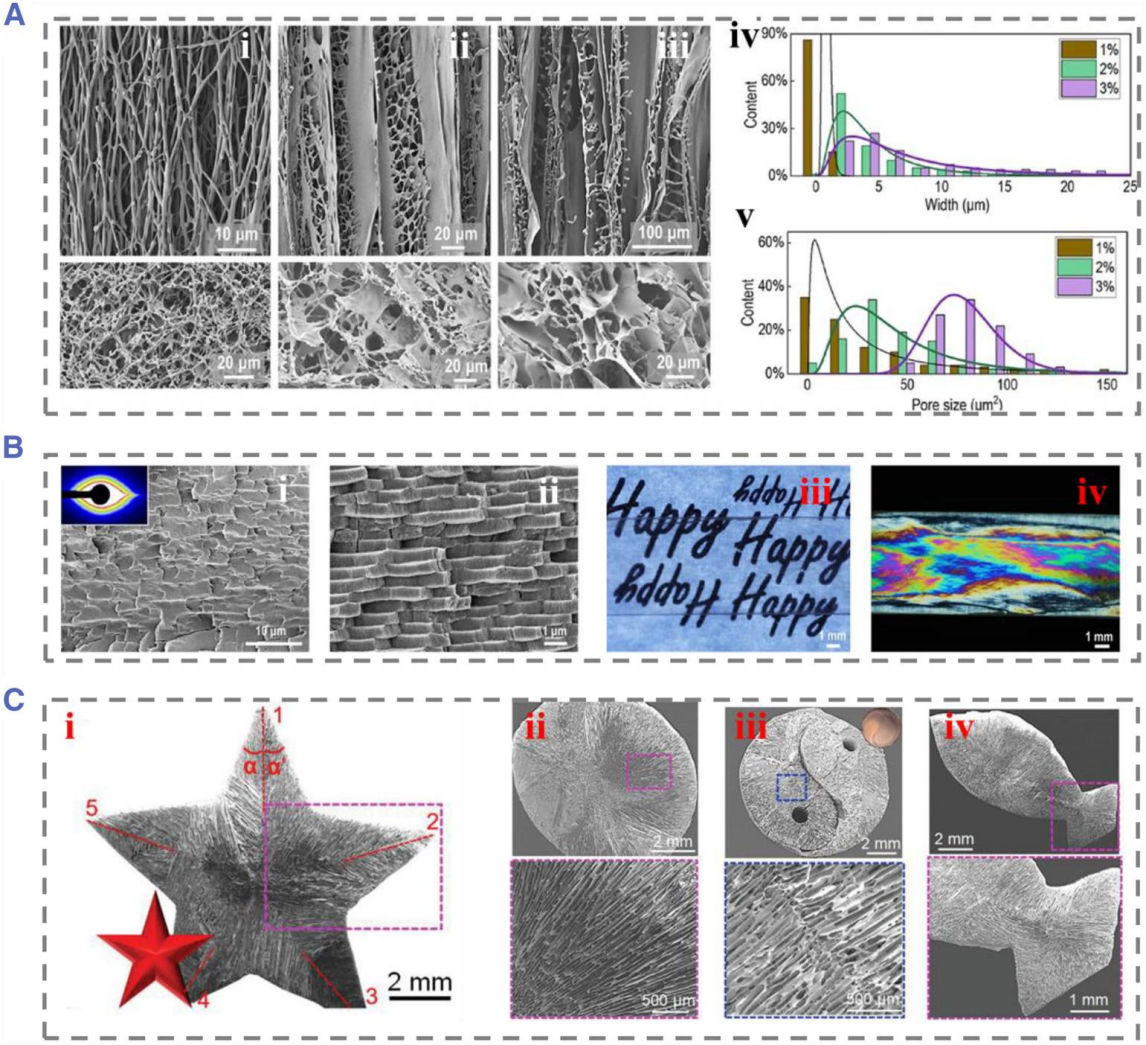
(A) (i–iii) SEM images of raw fiber and layered structure of foam prepared with silk fibroin solution of different concentrations. (iv) Distribution of wall thickness and (v) pore diameter of silk fibroin foam obtained using different silk fibroin solution concentrations. (B) SEM image of (i) cross section of silk fibroin nacre made by hot pressing silk fibroin foam, inner: 2D SAXS pattern of silk fibroin nacre layer. (ii) The physical microstructure of the natural nacre layer. (iii) The optical photos show the transparency of the silk fibroin nacre layer. (iv) The silk fibroin nacre layer exhibits birefringent color under polarized light. Reproduced with permission.^60^ Copyright 2022, Springer Nature. (C) (i) Pentagram structure copied with ice template. (ii) Circular, (iii) Tai Chi shaped, and (iv) fish structures prepared using programmable and template technology. Reproduced with permission.^61^ Copyright 2021, John Wiley and Sons. SEM, scanning electron microscopy.

Adjustable structural color micropatterns can be generated through the ice template method,^58^ and researchers have developed silk fibroin pearl layers to achieve high spatial resolution simulation of patterned surfaces from nanometer to micrometer scales^59^ (Figure 7A).^60^ Silk fibroin nacre boasts a microstructure similar to natural nacre but with enhanced strength and toughness that surpasses its natural counterpart. This enhanced mechanical resilience of silk fibroin nacre allows it to be processed using advanced techniques, including ion beam lithography. Moreover, silk fibroin nacre has a unique property of modulating the polarization of laser beams and generating structural colors (Figure 7B).^60^ The biocompatibility, processability, and tunable colorability of silk fibroin nacre make it a promising substitute for plastics in both structural engineering and biomedical applications. This promising material demonstrates the potential for clinical translation in the realm of implantable devices. Besides, multi‐directional freezing can also simulate the adjustable pore geometry of living organisms. Zhou et al. used ice template technology to control and program the local orientation of micropores (Figure 7C).^61^ They used specific copper molds to form temperature gradients from multiple field angles to form void structures with different multi‐dimensional orientations. This programmable technology enables the replication and manufacturing of three‐dimensional structures such as simulated starfish and fish.

### 4.3 Tissue regeneration scaffold

Tendons can serve as natural load‐bearing biomaterials to maintain strength and toughness.^62^ This remarkable resilience is attributed to the presence of a multi‐level structure in the tendon, where the hierarchical and functional structures are combined in a certain way.^63^ Hydrogels are fabricated through techniques such as electrospinning, extrusion, synthesis, cryocasting, self‐assembly, and mechanical stretching,^64^ with the aim of enhancing their mechanical properties.^65^ Nevertheless, numerous hydrogels with comparable water content fail to exhibit the superior strength, toughness, or fatigue resistance observed in tendons.^66^ He et al. introduced a multilength scale‐layered hydrogel structure through freeze‐assisted salting out.^67^ The PVA hydrogels obtained showed obvious anisotropy with micron‐sized cell pore walls and interlaced nanofiber networks. The water content of these hydrogels is more than 70%, which is similar to the human body. Their mechanical properties are similar to those of other solid hydrogels and even natural tendons. The proposed method is suitable for a variety of polymers, and it is possible to expand the use of structured hydrogels under the condition of requiring more stringent mechanical loading.

## 5 CONCLUSION

Ice templating is a versatile technique that enables the creation of materials with meticulously tailored porous structures, providing a unique opportunity to design and optimize materials to suit specific applications. In this comprehensive review, we explore and analyze previous methodologies employed in the fabrication of biomimetic structures, elucidating the exceptional porous characteristics exhibited by ice‐templated hydrogels in contrast to their uncrosslinked polymer solution counterparts. This groundbreaking approach has found extensive utilization across diverse fields including but not limited to tissue engineering and the development of intelligent bionic materials. The process entails the deliberate freezing of a suspension or solution under controlled conditions, followed by the subsequent removal of the ice, resulting in the formation of a highly porous structure. While this technology boasts numerous advantages and holds immense potential for various applications, it is crucial to acknowledge and address the existing challenges and limitations associated with its implementation.

Establishing a standardized protocol for the process of directional freezing poses significant challenges as each laboratory customizes devices with distinct settings, encompassing diverse models, configurations, and specification requirements. Typically, researchers resort to fabricate simplistic devices utilizing commonly available materials found in experimental laboratories, such as molds and liquid nitrogen. Ice‐templated biomaterials have exhibited remarkable versatility, fostering both commercial and clinical viability across numerous fields. To unlock the full potential of this technique for biomedical applications, a comprehensive understanding of the fundamental mechanisms governing polymer ice templates is imperative. Furthermore, unraveling the intricate relationship between pore characteristics and biological responses holds the key to advance the development of next‐generation ice template materials tailored specifically for biomedical applications. By delving deeper into these aspects, novel opportunities and pathways can emerge, leading to groundbreaking advancements in the realm of biomedical engineering.

Additional issues to be addressed include the time‐consuming and energy‐intensive freeze‐drying process, scalability, and reproducibility of production. In situ observations and a comprehensive understanding of ice growth are critical for addressing these questions. Recent studies have explored innovative approaches to overcome these limitations, such as room temperature thawing, ice‐dissolving complexation processes, and the study of colloidal particle motion and assembly during freezing. To tackle these challenges, in‐depth in situ observations and a comprehensive understanding of ice growth phenomena are essential. Recent studies have focused on innovative approaches to overcome these limitations. As our understanding of freezing mechanisms continues to advance and becomes more integrated with other complementary technologies, ice templating is poised to assume a more prominent role in the development of novel materials boasting exceptional properties. By addressing these challenges and exploring innovative methodologies, ice templating holds tremendous potential for revolutionizing the field of materials science and engineering.

## AUTHOR CONTRIBUTIONS

Hongbo Zhang conceived the idea and the proposal, Xiang Lin and Yunru Yu wrote the manuscript and edited the figure; Lu Fan, Li Wang and Anne M. Filppula revised the manuscript and assisted with the language; Hongbo Zhang supervised the manuscript.

## CONFLICT OF INTEREST STATEMENT

Hongbo Zhang is an executive editor for *Smart Medicine* and was not involved in the editorial review or the decision to publish this article. All authors declare that there are no competing interests.

## ETHICS STATEMENT

This review does not include human or animal research.
